# A comparative evaluation of the CN‐6000 haemostasis analyser using coagulation, amidolytic, immuno‐turbidometric and light transmission aggregometry assays

**DOI:** 10.1111/ijlh.13271

**Published:** 2020-06-17

**Authors:** Chris Gardiner, Philip Lane, Katy Langley, Hitesh Tailor, Samuel J. Machin, Ian J. Mackie

**Affiliations:** ^1^ Haemostasis Research Unit University College London London UK; ^2^ Haematology Evaluations Unit HSL (Analytics) LLP London UK

**Keywords:** coagulation, laboratory automation, laboratory practice

## Abstract

**Background:**

The CN‐6000 (Sysmex Corp.) is a new haemostasis analyser with blood coagulation, amidolytic, immuno‐turbidometric and light transmission aggregometry (LTA) capabilities. Transmitted light is monitored at multiple wavelengths (340, 405, 575, 660, 800 nm), from an LED light source.

**Aims:**

To evaluate the performance of the CN‐6000 against a predicate device.

**Methods:**

The CN‐6000 was evaluated against the CS‐5100 (Sysmex) for 14 different tests, using 880 samples from normal subjects, anticoagulated patients, critically ill patients, plasmas with high or low fibrinogen content or abnormal levels of interfering substances. Between‐day assay imprecision was assessed using commercial QC materials (n = 10 replicates on each of 5 days).

**Results:**

Acceptable levels of imprecision were obtained for all assays. Agreement between the two analysers was excellent for all assays. Throughput was 35% higher using the CN‐6000 (337 vs 250 tests per hour for PT, aPTT and fibrinogen). The CN‐6000 also demonstrated improved clot detection in plasmas with high levels of interfering substances as demonstrated by a 29% reduction in “vote‐outs” due to low light transmission (24 vs 34).

**Conclusions:**

The CN‐6000 demonstrated excellent comparability with the predicate instrument and acceptable levels of imprecision in all assays. Improvements in throughput and clot detection in the presence of interfering substances were also shown.

## 1 INTRODUCTION

Clinical laboratories are under increasing pressure to eliminate errors and reduce turnaround time to improve patient outcomes, while reducing costs. This is largely achieved through laboratory automation, with increasingly complex analysers performing an expanding range of tests but with reduced operator dependency. Haemostasis testing relies on several different technologies, and some specialist tests (eg platelet aggregation) have proved difficult to automate. The trend towards centralized multidisciplinary blood sciences laboratories and fewer specialist haemostasis technologists has driven the development of multi‐parameter haemostasis analysers capable of performing simultaneous testing using several methodologies.^1^ Many of these instruments routinely assess sample quality by checking for fill volume, optical interference by haemolysis, icterus and lipaemia (HIL checking). There has also been a drive towards smaller blood collection tubes and lower sample dead volume, which is necessary to avoid iatrogenic anaemia^2^ and is essential for analysing paediatric samples. Space in clinical laboratories is often limited, and in many core laboratories, it is essential that any new analyser can be incorporated into the laboratory's track system.

The CN‐6000 (Sysmex Corp) is a new high throughput, cap‐piercing haemostasis analyser with blood coagulation, amidolytic, immuno‐turbidometric and light transmission aggregometry capabilities. Transmitted light is monitored at multiple wavelengths (340, 405, 575, 660, 800 nm), from an LED light source, which combined with improved clot detection algorithms is intended to reduce sample rejection due to optical interference by HIL through wavelength switching and automatic high gain detection settings. It also has a relatively small footprint (CN‐6000; 720 × 906 × 1350 cm vs CS‐5100; 1030 × 1150 × 1280 cm) and can readily be incorporated into a track system.

The purpose of this study was to compare the performance of the CN‐6000 haemostasis analyser against the predicate device (CS‐5100 system) for assays using clotting, amidolytic, immunoturbidimetric and light transmission aggregometry (VWF ristocetin cofactor assays) methodologies, ^3^, ^4^, ^5^ using reagents recommended by the manufacturer (Table 1). The protocol was designed using published guidelines.^6^, ^7^ This included an assessment of precision, comparability, carryover, HIL checking and throughput.

**Table 1:** Reagents and calibrators used on the CN‐6000 and CS‐5100

Test	Reagent	Calibrators
Prothrombin time/INR	Dade^®^ Innovin^®^	AK Calibrant
Activated partial thromboplastin time	Dade^®^ Actin^®^ FS	Standard Human Plasma
Clauss fibrinogen	Dade^®^ Thrombin Reagent	Standard Human Plasma
Thrombin time	Test Thrombin Reagent	Standard Human Plasma
Factor VIII	Dade^®^ Actin^®^ FS FVIII deficient plasma	Standard Human Plasma
Factor XIII	Berichrom^®^ Factor XIII assay	Standard Human Plasma
Antithrombin	Innovance^®^ Antithrombin	Standard Human Plasma
D‐Dimer	Innovance^®^ D‐dimer	D‐Dimer Calibrant
VWF Activity	Innovance^®^ VWF Ac assay	Standard Human Plasma
VWF ristocetin cofactor activity	BC von Willebrand Reagent	Standard Human Plasma
Rivaroxaban (anti‐Xa activity)	Biophen™ Dixal Rivaroxaban	Rivaroxaban calibrator
Apixaban (anti‐Xa activity)	Biophen™ Dixal Apixaban	Apixaban calibrator
Edoxaban (anti‐Xa activity)	Biophen™ Dixal Edoxaban	Edoxaban calibrator
Dabigatran (Antithrombin activity)	Biophen™ DTI	Dabigatran calibrator

## 2 METHODS

Citrated plasma was obtained from residual, anonymized samples (collected into 0.109 mol/L sodium citrate (Vacutainer; Becton Dickinson) after all routine testing had been completed, in compliance with local ethical committee rules and the Human Tissue Act. Samples were frozen at −80°C and thawed at 37°C immediately prior to testing. Normal citrated plasma collected locally from apparently normal healthy volunteers and commercially sourced plasmas (CRYOcheck™ Normal Donor Set; Precision BioLogic Inc) were also tested. Informed consent was obtained from normal donors (approved by the UCL Research Ethics committee: Project ID Number: 7029/001). In total, 880 samples from normal subjects, patients receiving warfarin, low molecular weight heparin (LMWH) or direct oral anticoagulants (rivaroxaban, apixaban, edoxaban and dabigatran), as well as patients with critical illness, lupus anticoagulant, haemophilia A or von Willebrand disease (vWD), and samples with low or high fibrinogen levels, high levels of haemolysis, icterus or lipaemia were tested.

Due to limited clinical sample availability, citrated whole blood from several normal donors was spiked with unfractionated heparin (5000 IU/mL Multiparin; Wockhardt UK Ltd) before plasma preparation and dilution with autologous unspiked plasma to yield a range of concentrations. As dabigatran is seldom used in the UK, external quality control samples, quality control (QC) samples and normal plasma were used to prepare plasmas with a range of dabigatran concentrations.

Clotting tests (PT, APTT, fibrinogen and Factor VIII), amidolytic (factor XIII, antithrombin, anti‐Xa, direct thrombin inhibitor, and anti‐Xa DOAC), immuno‐turbidometric (VWF activity and D‐dimer) and light transmission aggregometry (VWF ristocetin cofactor assay) methodologies were evaluated.

Standard curve reproducibility was studied by performing calibrations on five different days using the reagents and calibrators shown in Table 1 (All reagents from Siemens Healthcare, Marburg, Germany). Imprecision was assessed by testing freshly reconstituted lyophilized quality control (QC) samples ten times on five separate days. The QC plasmas used for each test are listed in Table 2.

**Table 2:** Between‐day imprecision for the CS‐5100 and CN‐6000 analysers. 10 replicates of each control were performed on at least 5 different days

Test	Control	CS‐5100			CN‐6000		
		Mean	SD	CV (%)	Mean	SD	CV (%)
Prothrombin time (s)	Citrol 1	11.1	0.2	1.6	11.1	0.1	1.3
	Citrol 2	30.3	0.4	1.5	30.4	0.4	1.4
	Citrol 3	49.8	1.4	2.8	49.8	1.1	2.2
Activated partial thromboplastin time (s)	Citrol 1	24.9	0.2	0.9	25.0	0.3	1.1
	Citrol 2	46.0	0.8	1.6	46.2	0.8	1.7
	Citrol 3	72.5	0.7	1.0	72.4	0.7	0.9
Clauss fibrinogen (g/L)	Control N	2.83	0.11	4.1	2.72	0.14	5.0
	Control P	1.01	0.11	11.0	0.93	0.07	8.1
	High fbg pool	6.62	0.26	3.9	6.48	0.25	3.9
Thrombin time (s)	Control N	19.5	0.4	2.1	19.4	0.5	2.8
	Control P	25.4	1.9	7.6	25.1	1.0	4.0
	Heparin low control	33.6	4.4	13.2	33.8	4.1	12.1
Factor VIII (IU/dL)	Control N	93.3	2.2	2.3	91.6	2.7	3.0
	Control P	31.4	0.5	1.5	32.4	0.7	2.0
Factor XIII (IU/dL)	Control N	85.9	3.5	4.0	88.7	2.8	3.2
	Control P	31.0	2.2	7.2	31.9	1.4	4.5
Antithrombin (IU/dL)	Control N	95.8	1.0	1.1	96.6	1.2	1.3
	Control P	32.7	0.9	2.6	33.7	1.1	3.2
D‐dimer mg/L FEU	D‐dimer control I	0.30	0.02	5.5	0.32	0.07	3.2
	D‐dimer control 2	2.59	0.17	6.5	2.76	0.07	3.2
VWF activity (IU/dL)	Control N	101.3	6.7	6.6	103.8	4.6	4.5
	Control P	28.3	2.0	6.9	28.6	1.5	5.3
VWF ristocetin cofactor	Control N	88.9	8.1	9.0	87.3	7.6	8.8
(IU/dL)	Control P	24.4	2.4	9.8	24.8	2.4	9.6
Rivaroxaban (ng/mL)	Rivaroxaban C1	111.8	3.9	3.5	111.8	4.4	3.6
	Rivaroxaban C2	325.7	4.8	1.5	330.3	8.8	2.6
Apixaban (ng/mL)	Apixaban C1	354.7	24.7	7.0	373.0	20.2	5.4
	Apixaban C2	158.9	7.6	4.8	167.7	9.3	5.5
Edoxaban (ng/mL)	Edoxaban C1	329.3	7.7	2.4	333.3	8.8	2.6
	Edoxaban C2	158.9	7.6	4.8	167.7	9.3	5.5
Dabigatran (ng/mL)	Dabigatran C1	317.5	5.0	1.6	308.5	9.6	3.1
	Dabigatran C2	106.1	4.4	4.4	105.8	5.7	5.4

For the comparability study, samples were tested on the CS‐5100 and CN‐6000 within the same 2‐hour period. The same reagents and redilution limits were used on both instruments. FVIII assays were performed in multidilution analysis mode (MDA) using low normal or high dilution ranges to ensure that the clotting times were on the linear part of the calibration curve, and the mean of the three measured FVIII concentrations was reported. Von Willebrand factor (VWF:Ac) activity was measured using normal (25 ‐ 600 IU/mL) or low (0‐25 IU/mL) calibration curves as appropriate. The dilution used to assay von Willebrand ristocetin cofactor (VWF:RCo) was guided by the VWF:Ac result.

Cholesterol and triglycerides were measured using Chol2 and triglyceride reagents using a Cobas C111 analyser (Roche Diagnostics). Plasma haemoglobin was measured using the Hemocue system (Radiometer).

Sample and reagent carryover were investigated as previously described^6^ Briefly, sample carryover was assessed by performing APTT testing on aliquots of normal plasma (A1‐A3) and plasma containing 1 IU/mL unfractionated heparin (B1‐B3) in the sequence A1, A2, A3, B1, B2, B3 ten times, then repeating the sequence in reverse. Reagent carryover was investigated by performing APTT testing on five aliquots of normal plasma before performing Clauss fibrinogen assays on the same five aliquots. This sequence was repeated ten times.

Statistical analysis was performed with GraphPad Prism version 8.0.0 for Windows (GraphPad Software). Deming regression analysis and Bland Altman analysis were performed to assess comparability.

## 3 RESULTS

The between‐run coefficient of variation (CV) for calibration curve imprecision for PT, Clauss fibrinogen, D‐dimer, antithrombin, dabigatran and FVIII assays was <3.0%. The CVs for the calibration curves for rivaroxaban, apixaban and edoxaban anti‐Xa assays were all <5%. Between‐run calibration curve CVs for VWF:Ac and VWF:RCo were <10% and <15%, respectively.

The between assay imprecision results are shown in Table 2. In all cases, the %CV values for both instruments were lower than the manufacturer's specifications for within‐run imprecision for a normal control plasma for the CS‐5100.

The performance of the HIL checking function was assessed by testing 40 normal, 30 icteric, 30 haemolysed and 40 lipaemic samples. Lipaemia was defined as triglycerides of >1.88 mmol/L or cholesterol of >5.2 mmol/L, haemolysis as plasma Hb of >0.5 g/L, and icterus as bilirubin >17 µmol/L. As very high levels of any interfering substance will trigger all three HIL flags, sensitivity and specificity was assessed by testing the ability of the analysers to trigger HIL flags in the presence of lipaemia, haemolysis or icterus. The sensitivity of both instruments to HIL was 96.9%. The CS‐5100 generated HIL flags in the absence of any HIL in 4 samples while the CN‐6000 had only one false flag, resulting in specificity of 90.7% and 97.7%, respectively.

**Figure 1:**
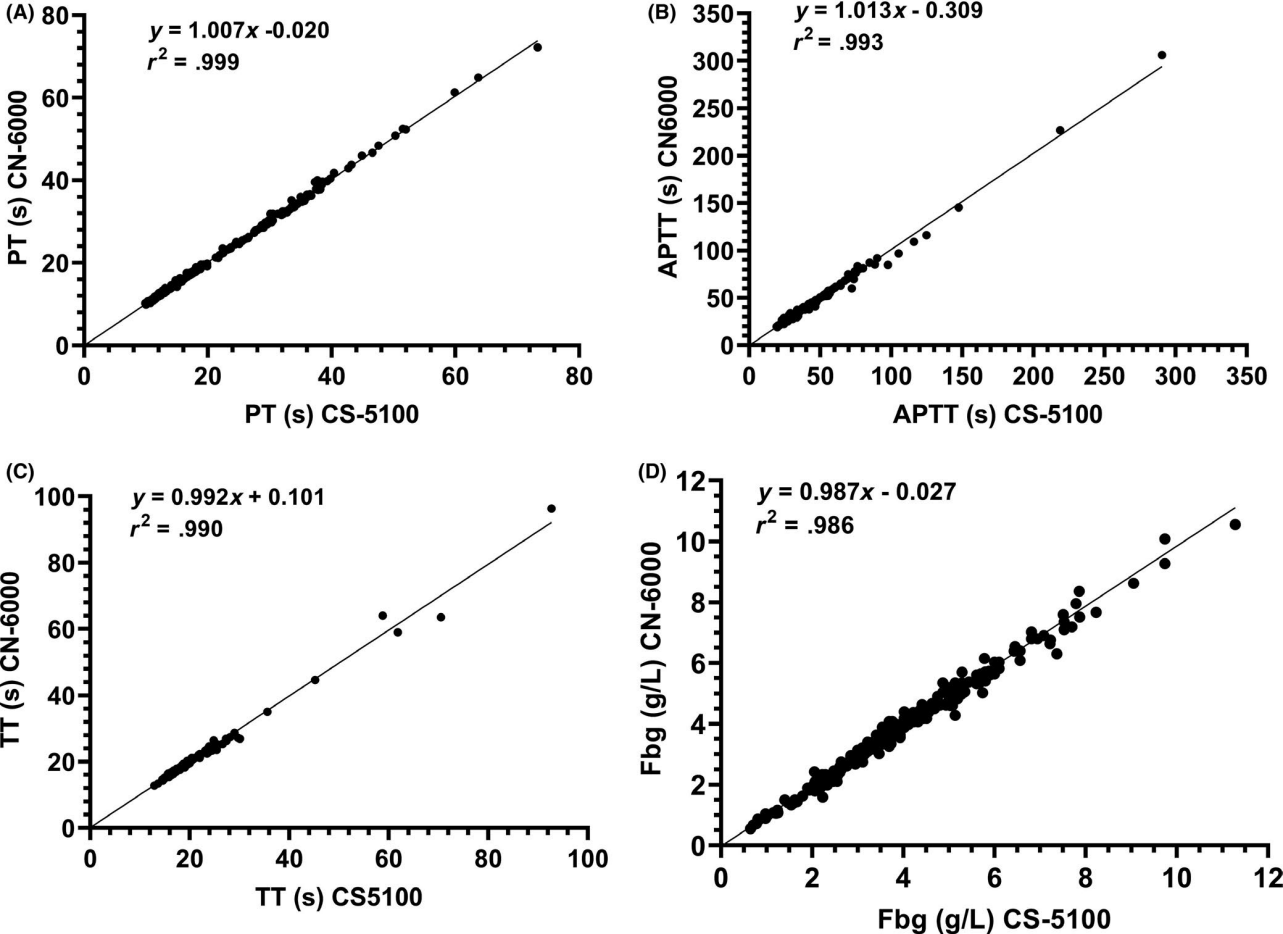
Regression graphs for CS‐5100 vs CN‐6000. A, prothrombin time, B, activated partial thromboplastin time, C, thrombin time, D, Clauss Fibrinogen

**Figure 2:**
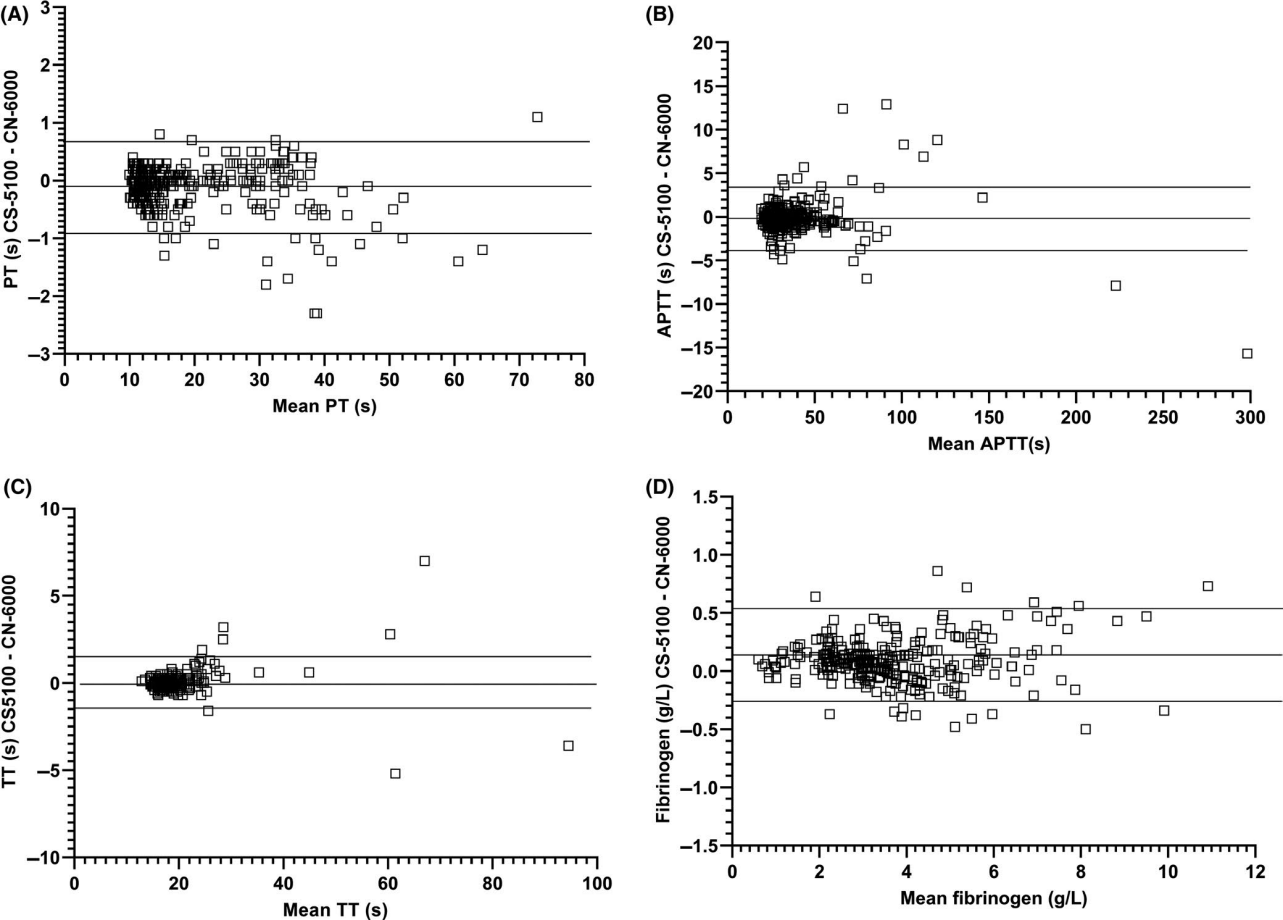
Altman Bland plots for A, prothrombin time, B, activated partial thromboplastin time, C, thrombin time, D, Clauss Fibrinogen

Comparability for PT was assessed in 389 samples including normal plasmas, plasma from patients receiving warfarin and DOACs, and plasmas with high levels of HIL. Excellent agreement was obtained between the methods with no obvious trend with increasing PT (Figures 1 and 2, Table 3). 10/40 and 6/40 lipaemic samples failed to give PT results with the CS‐5100 and CN‐6000 analysers, respectively (triglycerides 4.1 ‐ 19.6 mmol/mL, cholesterol 4.01‐9.68 mmol/L). All samples with triglyceride concentrations of <8 mmol/L gave PT results on the CN‐6000. Two of 30 haemolysed samples with plasma haemoglobin levels of 8.4 and 19 g/L failed to give results with either instrument. No optical interference due to icterus was observed in PT performed on 30 icteric samples (bilirubin 20‐493 µmol/L). Clauss fibrinogen assays performed on 327 of the same plasmas also showed good agreement. Optical interference occurred in two lipaemic samples (triglycerides >10.0 mmol/L) with both instruments, but no interference due to icterus or haemolysis was observed.

**Table 3:** Regression parameters and correlation coefficients for CS‐5100 vs CN‐6000. Only samples giving results with both instruments were used

Test	n	Slope	Intercept	R^2^	Mean CS‐5100	Mean CN‐6000	Range CS‐5100
Prothrombin time (s)	374	1.007	−0.020	0.999	18.51	18.58	9.9‐73.3
APTT (s)	362	1.013	−0.309	0.993	32.74	32.93	19.3‐290.4
Thrombin time (s)	252	0.992	0.101	0.990	19.57	19.53	12.9‐92.7
Clauss fibrinogen (g/L)	325	0.987	−0.027	0.986	3.74	3.67	0.65‐11.28
Factor VIII (IU/mL)	204	0.996	0.136	0.996	108.0	107.3	1.5‐456.0
Factor XIII (IU/mL)	165	0.969	4.343	0.971	87.3	89.2	1.3‐183.3
Antithrombin (IU/mL)	197	1.037	−2.182	0.997	83.3	83.5	18.6‐148.9
D‐dimer mg/L FEU	234	0.898	0.059	0.993	1.75	1.86	0.19‐25.75
VWF activity (IU/mL)	148	0.977	4.298	0.994	150.8	151.7.	4.5‐538.9
VWF ristocetin cofactor (IU/mL)	123	0.976	3.530	0.887	105.4	106.4	12.1‐274.4
Rivaroxaban (ng/mL)	28	0.982	1.693	0.994	183.1	181.4	39.4‐425.4
Apixaban (ng/mL)	25	0.901	8.345	0.973	157.3	150.1	60.4‐264.9
Edoxaban (ng/mL)	12	1.073	−37.0	0.986	203.4	181.3	97.4‐475.0
Dabigatran (ng/mL)	28	0.960	9.27	0.994	258.8	247.2	60.7‐532.0

Thrombin time comparability was assessed in 259 samples (including normal plasmas, plasmas with high levels of D‐dimer and HIL). Good agreement was observed between methods. Optical interference was observed in seven lipaemic samples (triglyceride >8.0 mmol/L) on both instruments. Haemolysis (plasma Hb 0.5‐19 g/L) and icterus (bilirubin 20‐493 µmol/L) did not cause optical interference.

APTT measurements performed on 362 samples including plasmas from normal subjects, patients receiving LMWH, UFH or DOACs, patients with haemophilia, vWD, or lupus anticoagulant, and plasmas with high levels of HIL also showed excellent agreement. No optical interference was observed in 40 lipaemic plasmas (triglyceride 1.4‐12.4 mmol/L, cholesterol 3.9‐9.78 mmol/L), 30 icteric plasmas (20‐265 µmol/L) or 30 haemolysed plasmas (plasma Hb 1.3‐25.6 g/L).

A range of plasmas were used to assess comparability for FVIII, FXIII, vWF:Ac, vWF:RCo, DOACs, D‐dimer and antithrombin (Table 3). Only samples giving results within the reportable range for both instruments were used for the analysis. Consequently, although large numbers of samples (180‐300) were tested, some tests produced many results outside the reportable range (eg DOAC and. VWF:RCo) and regression analysis was performed on a relatively small number of results. Despite this, *r* ^2^ values of >0.9 were reported for all tests except VWF:RCo (Table 3).

Throughput was assessed by testing PT, APTT and fibrinogen in 60 plasma samples representative of a typical hospital laboratory workload. Testing was performed in cap‐piercing mode, with automatic reflex testing for samples with fibrinogen results outside the redilution points (<0.8 g/L and > 4.5 g/L). The throughput of the CN‐6000 was 337 test/hour versus 250 test/hour for the CS‐5100. No reagent or sample carryover was detected.

## 4 DISCUSSION

The purpose of this study was to compare the performance of the CN‐6000 haemostasis analyser with the established CS‐5100 system for assays using clotting, amidolytic, immunoturbidimetric and light transmission aggregometry (VWF ristocetin cofactor assays) methodologies. Intra‐assay imprecision for both analysers was within the manufacturer's specification for the CS‐5100 and similar to previous studies of the CS‐5100.^3^, ^4^, ^5^ Agreement between the two analysers was excellent for the 14 assays studied in a wide range of samples. Optical interference was observed in some tests in a few samples with exceptionally high levels of lipaemia (triglyceride > 8 mmol/L) and haemolysis (plasma Hb > 8.4 g/L). When errors related to the amount of transmitted light (Turbidity Level Over, Trans Light High, No Coagulation and Slight Coagulation) due to the influence of interfering substances (eg HIL) are generated, the CN‐6000 automatically switches to high gain. If the errors are still present, the detection wavelength is switched for clotting tests only, for example PT 660‐800 nm. This resulted in a slight reduction in samples for which results could not be obtained, with the CN‐6000. All samples with HIL levels within the manufacturer's specified limits gave results.

HIL flagging was assessed in 140 samples. Although HIL flagging is only an approximation of haemolysis, icterus and lipaemia based on light transmission, both instruments demonstrated a sensitivity of 96.9% to optical interference due to HIL. However, the CN‐6000 showed improved specificity to HIL (97.7% vs 90.7% for the CN‐6000 and CS‐5100, respectively). Importantly, haemolysis and lipaemia were flagged at levels previously reported to cause clinically important discrepancies in some coagulation tests.^8^, ^9^, ^10^ No sample or reagent carryover was detected, and the throughput of the CN‐6000 was 35% higher than the CS‐5100 in sample representative of a normal hospital laboratory workload. This is the result of an improved high‐pressure rinsing system, which reduced the time for pipette rinsing.

We did not evaluate LTA on fresh platelets in this study, as quantitative comparative evaluation of LTA is extremely difficult. There are no standards, no quality control materials and fresh platelets are labile. For this reason, VWF:RCo with freeze‐dried platelets was selected to evaluate the LTA measurement feature on the CN‐6000. However, we acknowledge that formalin‐fixed platelets do not behave in the same way as fresh platelets, and this is a limitation of this study.

In conclusion, the CN‐6000 demonstrated excellent comparability with the predicate instrument and acceptable levels of imprecision in all assays, with improved throughput and HIL detection.

## CONFLICT OF INTEREST

The work was supported by an institutional, unrestricted research grant from Sysmex UK Ltd. However, Sysmex had no part in the data analysis or preparation of the manuscript. CG, IJM and SJM are consultants for Sysmex Corp.

## AUTHOR CONTRIBUTIONS

CG designed the study, performed assays, analysed the data and wrote the manuscript. PL, KL and HT performed assays and analysed data. SJM and IJM designed the study, aided in data analysis and critically reviewed the manuscript.
